# Co-development, initial evaluation and implementation of a digital health intervention (SmartADHD) for young people (aged 16–30) with attention deficit hyperactivity disorder, including optimisation for sustainable delivery via health and social care settings: protocol for a mixed-methods study

**DOI:** 10.1136/bmjopen-2026-127810

**Published:** 2026-10-20

**Authors:** Anna Price, Eleanor Bryant, James Wenger, Madeleine J Groom, Helen Atherton, Tamsin Newlove-Delgado

**Affiliations:** 1 Health and Community Sciences, University of Exeter Medical School, Exeter, Devon, UK; 2 Institute of Mental Health, University of Nottingham Faculty of Medicine and Health Sciences, Nottingham, UK; 3 Primary Care Research Centre, University of Southampton, Southampton, UK; 4 Public Health and Sport Sciences, University of Exeter Medical School, Exeter, UK

**Keywords:** Primary Care, Attention Deficit Disorder with Hyperactivity, Young Adult, QUALITATIVE RESEARCH, Digital Technology

## Abstract

**Abstract:**

**Introduction:**

Attention deficit hyperactivity disorder (ADHD) is a neurodevelopmental condition affecting 5–7% of children/adolescents and 2–5% of adults. It is characterised by inattention, hyperactivity and impulsivity, impacting functioning across multiple domains with linked social and economic burden. Despite effective treatments that reduce the risk of negative outcomes, the UK is currently failing to provide equitable access to healthcare for people with ADHD. Young people with ADHD are at a vulnerable life-stage, facing multiple transitions such as moving educational settings, starting work or leaving home. They frequently experience barriers to accessing care, including unsupported healthcare transitions, service gaps and attentional/organisational difficulties. A co-developed digital health application (app) could provide cost-effective psychoeducation and self-management tools for young people aged 16–30. This includes support at key times when standard services may not be available, providing trusted healthcare information and helping them to navigate care pathways, ‘wait well’ for referral/assessment or their next appointment and manage this long-term condition. This mixed-methods study aims to co-develop, initially evaluate and plan the implementation of a digital health intervention (the SmartADHD app) for young people with ADHD.

**Methods and analysis:**

This mixed methods research will follow the Person-Based Approach to co-develop, iteratively adapt and feasibility test a prototype digital health intervention, informed by a critical realist stance. An interdisciplinary team, comprising experts by lived experience, health and social care professionals and digital experts, will follow co-production and engagement methodologies to develop theory, prioritise content and co-design a simple prototype (V. 0). Work Package 1 will trial and refine the prototype with 20 young people via community settings to establish usability and acceptability (V.1). Work Package 2 will feasibility test the refined prototype with 30 young people and eight healthcare professionals in National Health Service (NHS) settings (V.2). Data will be collected via think-aloud interviews, semi-structured interviews and pre- and post-surveys to iteratively improve and initially evaluate the prototype. Concurrent workstreams will explore economic models for long-term NHS implementation and address digital inclusion.

**Ethics and dissemination:**

The protocol has been approved by the Yorkshire and The Humber - Sheffield Research Ethics Committee (26/YH/0101). Recruitment will commence in September 2026. Young people with lived experience of ADHD will be actively involved as co-authors and co-presenters in communicating outputs. All participants will provide informed consent prior to participation. Findings will be disseminated via research articles in peer-reviewed journals, conference presentations, public involvement events, patient groups and media releases. A summary of study findings will be shared with participants at the end of the study.

**Trial registration number:**

ISRCTN26360329.

**STRENGTHS AND LIMITATIONS OF THIS STUDY**

- The SmartADHD app is aimed at young people aged 16–30, a group who are at risk of experiencing gaps in care between child and adult services.
- Sampling from community and primary care settings will reach a range of participants, and young people who are on the waiting list for attention deficit hyperactivity disorder (ADHD) diagnosis are not excluded from participating.
- The app has been co-developed by young people, with content informed by a consultation exercise of over 40 people, and the present study will take a multi-disciplinary approach informed by the lived experience, healthcare professional and digital research advisory groups (RAGs).
- Preliminary implementation work will investigate the views of healthcare practitioners, ensuring it is tailored to their needs, and unlike many commercially available ADHD apps, SmartADHD will aim to meet NHS quality standards and integrate within existing NHS pathways.
- The opportunistic sample may introduce selection bias and limit the generalisability of the study findings, and the use of digital technology may risk digital exclusion and widening health inequalities among some groups.

## Introduction

### Background

 Attention deficit hyperactivity disorder (ADHD) affects 5–7% of children and adolescents and 2–5% of adults globally.^1^ It is a complex, highly heritable neurodevelopmental condition that often co-occurs with other conditions, such as autism, and commonly persists across the lifespan.^1 2^ ADHD is characterised by hyperactivity, impulsivity and/or inattention that interfere with daily functioning. ADHD impacts multiple life domains including physical and mental health, relationships, work and education, with high costs for individuals and society.^3^

The economic burden of ADHD is substantial, with global annual costs in 2021 estimated at £637–£16 300 per person.^4^ UK annual costs to the NHS, Social Care and Education are estimated at £8200 per adolescent (adjusted for inflation).^5^ Higher ADHD prevalence is linked with economic disadvantage, and the costs to families raising children with ADHD are estimated to be five times higher than for children without the condition.^6 7^ Despite effective treatments which reduce risks of negative outcomes,^1 2^ multiple barriers prevent young people with ADHD from accessing health and social care. These include communication issues, attentional challenges and organisational difficulties, exacerbated by gaps in specialist provision, unsupported transitions between child and adult services, long waiting lists and confusing care pathways.^8–11^ NHS England’s recent Task Force for ADHD acknowledged these challenges and identified the need to improve care through a joined-up approach.^12^ Furthermore, the UK Government’s 10 Year Health Plan sets out a vital shift towards digitally enabled, preventative and community-based care, emphasising the use of new technologies to improve access and outcomes.^13^ The Taskforce report specifically urged government to include ADHD health services in its 10-year plan for digitisation, emphasising that ADHD services need to be digitised, more efficient and supported by better data infrastructure.^14^

### Need for innovation

Cost-effective innovations for managing ADHD are urgently needed, given stretched NHS and Social Care resources,^11^ increasing mental health needs among young people and longer waiting lists for assessments and treatment since the COVID-19 pandemic.^15^ A digital health intervention (DHI), such as an app, providing targeted healthcare information, psychoeducation and self-management support, could be prescribed via primary or secondary care, providing a scalable solution to support patients when services are hard to reach.^2 16^ Research conducted by our research collaboration has identified that healthcare apps are the most desired yet least used information resources for patients, primary healthcare professionals and commissioners.^17^ The current context of multiple unregulated and untrustworthy digital resources creates confusion for patients and providers.^18 19^ A rational, evidence-based approach is needed to co-develop an app that is trusted by patients and providers and improves access to healthcare for young people with ADHD by helping them to understand their condition and the different ways services provide assessment and treatment. This app needs to be co-developed with experts by experience to ensure it is engaging to ‘ADHD brains’, accessible to underserved groups, such as care leavers and available ‘24/7’ to overcome time-management issues. It must be designed to fit into existing systems, helping young people access care and ‘wait well’ for referral/assessment or their next appointment.

This study builds on our team’s extensive research into healthcare provision for young people with ADHD,^9 16 17 20–22^ findings from our mixed-methods systematic review of DHIs for young people with ADHD^23^ and training and development work funded through the lead author’s National Institute for Health and Care Research (NIHR) Development and Skills Enhancement Award, to co-develop such an intervention.

### Review of prior evidence

Advances in digital mental health have led to the development of DHIs, which can reduce consultation time, shorten waiting lists, promote self-care and be highly cost-effective due to scalability.^24^ National Institute for Health and Care Excellence (NICE) now conditionally recommends several DHIs, such as the ‘Silvercloud’ app for anxiety and depression, demonstrating their ability to support well-being in young people. However, evidence-based DHIs for neurodevelopmental conditions are still in early stages of development, with limited evidence of effectiveness.^25^

A 2017 systematic review found some evidence of clinical benefit from DHIs for depression and anxiety in young people, but benefits for ADHD were uncertain.^26^ A 2019 meta-analysis of web-based interventions for children and young people with neurodevelopmental conditions found some evidence of effectiveness in improving condition-specific outcomes, like time-management skills and reducing comorbid psychological symptoms, such as anxiety.^27^ However, methodological limitations were highlighted. Our systematic review^23^ of DHIs providing healthcare information and self-management resources for people aged 16–25 with ADHD identified only five randomised controlled trials, none from the UK and only one positioned in primary care settings.^28^ Promisingly, this Norwegian study of a co-developed intervention demonstrated improvements in self-reported ADHD symptomatology and quality of life through the use of a DHI.^28^ Together with the success of the ‘Silvercloud’ app, this suggests that, with appropriate stakeholder-informed development, a curated evidence-based app could similarly improve outcomes for people with ADHD in the UK.

Despite the growth in digital mental health, methodological guidance for research and implementation of digital approaches is still lacking.^29^ Benefits are not accessible to everyone, with certain groups, such as those with limited formal education, at risk of digital exclusion, potentially exacerbating health inequalities. Updated Medical Research Council (MRC) guidelines for developing complex interventions reflect recent theoretical and methodological advancements,^30^ but more specific guidance is needed for DHIs targeting individuals with neurodiverse health needs. Key considerations when developing apps for young people with ADHD include using agile user-centred design and co-production,^31^ involving interdisciplinary teams^32^ and including people from underserved backgrounds. Planning for implementation and demonstrating acceptability, feasibility and effectiveness are also crucial.^26^

Evidence-based approaches to developing apps for this group include ensuring content and modes of delivery are co-designed with experts by experience to account for attentional issues and ensure acceptability and usability^33^ and using user-centred methodologies underpinned by theory when co-developing, feasibility testing and evaluating DHIs.^33^ Emerging evidence indicates that a successful app for young people with ADHD would include features such as digital psychoeducation with self-management support, patient stories and healthcare reminders.^18 27 33 34^ These features have the potential to improve ADHD symptoms, reduce anxiety and depression and enhance quality of life.^27 28^

### Rationale

Transition from child to adult services occurs during a critical period for young people with ADHD, yet it is poorly supported.^8 9^ ADHD is underdiagnosed and under-treated in young people,^6 10 11 35^ with worse healthcare access for some groups such as individuals from deprived areas.^6 35^ Equality, diversity and inclusion (EDI) issues are intensified by increasing demands on services.^11^ Young people with ADHD, particularly those aged 16–30, are at increased risk of losing contact with specialist services, experiencing mental health crises and developing long-term psychiatric conditions.^1 8 22^ Our 2019 mixed methods study found that only 22% of UK youths aged 17–19 needing transition to adult ADHD services attended their first appointment, with just 6% meeting optimal transition criteria.^8^ People aged 18–25 can wait over 2 years for an ADHD assessment for a potential diagnosis, with little to no support.^10 11 16^ Transition occurs at a crucial time for long-term condition management when patients may be undertaking key exams or starting work.^8^ Even after diagnosis, service gaps and long waiting lists mean essential healthcare information, psychological therapies and mental health support are commonly unavailable despite being recommended in the NICE guidelines.^36^ Co-production research conducted with our team has shown that challenges persist past the age of 25, indicating that interventions need to be designed for and with young people aged up to the age of 30.^37^ This research focuses on young people aged 16–30 years to address long term condition management into adulthood, but may also benefit others. This is in line with research indicating that the second major phase of brain development across the lifespan completes around the age of 32.^38^

Primary care providers (eg, general practitioners (GPs)) are the interface between patients and specialist services in the UK. They are typically the first and sometimes only consistent healthcare contact for young people with ADHD.^2^ GPs also have a role to play in managing broader mental and physical healthcare, such as for co-morbid conditions such as depression or diabetes.^2^ Under UK ADHD guidelines, primary care professionals (eg, GPs) can refer patients for assessment and prescribe ADHD medication under shared care agreements.^36^ However, many feel unsupported to provide advice/healthcare and lack understanding of ADHD.^8^ With rising demand and limited resources, GPs are increasingly left to manage patients awaiting assessment/treatment without specialist input.

A DHI, such as an app, providing targeted healthcare transition information, self-management support and psychoeducational materials, could be ‘prescribed’ by GPs, social prescribers, specialist nurses or other healthcare professionals. This would enable primary care professionals to support the transition and long-term condition management of ADHD, without extensive additional training. The app could also offer early intervention support for those awaiting assessment or with sub-threshold symptoms,^15^ helping patients to manage their condition while waiting for specialist care.

### Aims and objectives

The overall aim of this research is to build on available evidence to co-develop, pilot and initially evaluate a digital health app for people aged 16–30 with ADHD, including optimisation for long-term implementation. The specific research objectives are to:

1. Investigate the required content, design and delivery mechanisms for a digital psycho-educational and self-management app for young people with ADHD (prototype development).
2. Assess the usability and acceptability of the co-designed prototype (proof of concept).
3. Evaluate the feasibility of delivering the app via NHS settings (feasibility study).

Additional secondary objectives are to:

- Prepare for a subsequent full-scale effectiveness-implementation trial in NHS settings.
- Explore digital inclusion factors and meet the health needs of underserved communities, such as socio-economically disadvantaged groups.

## Methods and analysis

### Overview

This mixed-methods research follows the updated MRC framework for developing and evaluating complex interventions and is informed by a critical realist stance.^30 31 39^ It consists of an initial phase of patient and public involvement and engagement (PPIE), followed by two work packages (WPs 1 and 2) delivered alongside ongoing content co-production and prototype software development. Implementation and EDI workstreams will run throughout, exploring sustainable NHS implementation and addressing digital inclusion barriers. For an overview, see figure 1.

**Figure 1:**
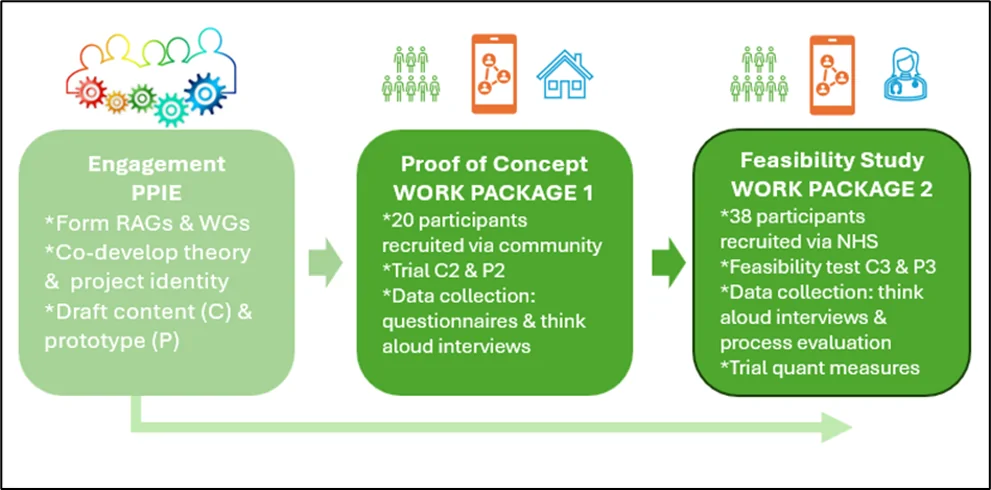
Research timeline. PIPE, Public Involvement and Engagement; RAG. Resarch Advisory Group; WG, Working Group.

#### PPIE (engagement)

Research partners will work alongside the core research team to build on existing evidence to co-develop theory, prioritise functional content and explore user experience (UX) preferences for the SmartADHD web-app. This will lead to co-developed versions of content (C) and a wireframe prototype (P) generated within Figma.^40^ PPIE engagement via study research advisory groups (RAGs) and working groups (WGs) will continue across the project lifecycle, with RAG meetings occurring every 6 months and WGs convened as needed.

#### Work package 1 (WP1; proof-of-concept)

WP1 involves 20 young people (either diagnosed with ADHD or actively awaiting assessment) and their supporters testing iterations of content (C1) and the prototype (P1) in community settings. Data collection will be via pre- and post-surveys, think-aloud interviews and semi-structured interviews/focus groups. Findings will directly inform finalised versions of C1 and P1 while providing preliminary evidence on system usability and acceptability.

#### Work package 2 (WP2; feasibility)

WP2 involves 30 NHS patients aged 16–30 with ADHD (or awaiting assessment) trialling iterations of content (C2) and the prototype (P2) in NHS settings. Eight healthcare professionals with experience signposting patients to the SmartADHD resource will also be involved. Patients will be ‘prescribed’ the app and prompted to access it over a 4-week period. Data will be gathered via pre- and post-questionnaires, think-aloud sessions, semi-structured interviews and automated backend records of time spent on prototype sections.

#### Study status and timeline

PPIE engagement commenced in November 2025, building on previous co-development activities and relationships, and will continue through the project. Work Packages 1 and 2 will run sequentially, with participant recruitment for WP1 opening in September 2026. Data collection for WP1 is expected to be completed by February 2027. Data analysis is expected to conclude by May 2028. Site identification for WP2 will commence in June 2027, with participant recruitment opening in September 2027. Data collection is planned for completion by April 2028. Final analysis and prototype refinement for WP2 are expected to conclude by December 2028. Implementation and EDI workstreams will be active throughout, with focused implementation activities commencing in January 2029 and completing in March 2030.

### Study design

#### Work package 1

A single-arm proof-of-concept study^41^ will follow the Person-Based-Approach,^31^ to evaluate user engagement, usability and acceptability of P1, while trialling outcome measures. WP1 incorporates a structured cycle of iterative refinement, leading to locked-down versions of P1 and C1. A subset of participants will be invited to participate in semi-structured interviews/focus groups to capture qualitative feedback regarding UX of engaging with the prototype.

#### Work package 2

A mixed-methods feasibility study will evaluate:

1. The acceptability and usability of engaging with P2 when prescribed (or signposted) via a clinician or study representative.
2. The feasibility of recruitment and data collection for a subsequent trial.
3. Participant feedback on the intervention.

Findings will inform the design of a pilot and hybrid effectiveness-implementation trial of the web-app.^42^ Feedback will inform iterative refinement of C2 and P2, culminating in a detailed technical specification for the SmartADHD web-app. A nested process evaluation will explore NHS implementation.

### Sampling and eligibility

The research team will purposively recruit a sample with a diversity of characteristics and experiences (eg, representing varied age categories, co-occurring conditions, ethnicity and time since diagnosis or waiting for assessment). The research team will aim to mitigate the risk of non-genuine participants by using strategies such as advertising via known networks and speaking to participants face-to-face.^43^

- **WP1**: 20 young people aged 16–30 with ADHD (10 with a diagnosis, 10 awaiting assessment). A subset (n=8) will complete qualitative interviews.
- **WP2**: 30 young people aged 16–30 with ADHD (15 with a diagnosis, 15 awaiting assessment) via NHS or community settings. Eight healthcare professionals (including clinical and administrative roles) with experience supporting people with ADHD (or suspected ADHD). A subset (n=up to 20) will complete think aloud interviews, another subset (n=14) will complete qualitative interviews.

For details see figure 2.

**Figure 2:**
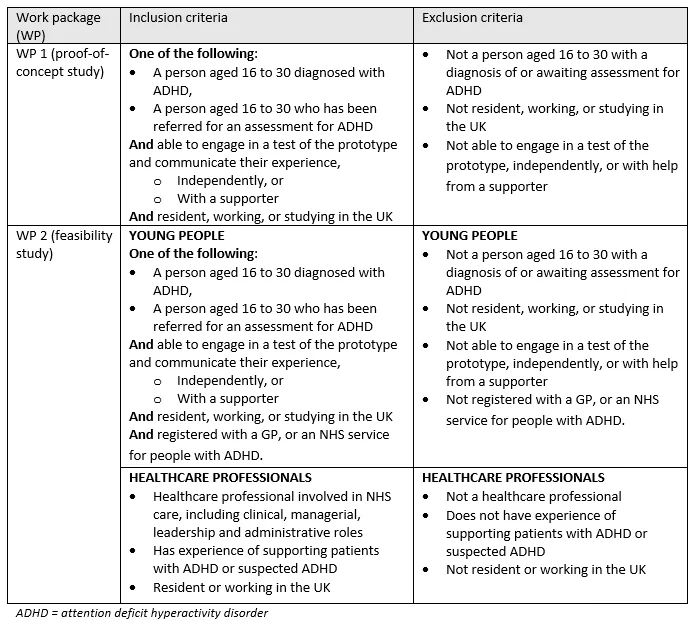
Participant eligibility criteria, by work package.

### Procedures

Young people will be invited to involve a supporter to help them to engage with the research. All participants will be offered a £30 gift voucher as a thank you for taking part, with healthcare professionals offered a certificate recognising their participation in research.

#### WP1

Participants will be purposively recruited from community settings using convenience sampling techniques (using social media/email) and via study and research partner networks (eg, MindTech, SAND, UK Adult ADHD Network) previously used by this team.^17^ They will be invited to complete an expression of interest form, and if selected, consented into the study. As detailed above, strategies will be used to mitigate the risk of non-genuine participants.

Consented participants will be invited to group workshops or one-to-one sessions in which they will trial P1. Workshops/sessions will be hosted in-person or virtually depending on participant preference and geographic location, with up to eight participants per workshop. Participants will complete pre- and post-questionnaires using secure online survey software (eg, Qualtrics). They will complete a comprehensive ‘think-aloud’ interview while actively navigating the prototype, where they will verbalise their thoughts, feelings, and behavioural actions; providing feedback on usability and real-time interactions.

User journeys will be tracked via secure UX tools natively connected to the Figma prototype (eg, Maze or UXtweak). Analysis of think-aloud data^44^ will inform iterative updates to P1 and C1. A sub-sample (n=8) will provide qualitative data on their experiences of engaging with the prototype, and research involvement, via semi-structured interviews/focus groups (online/in-person, approximately 60 min).

#### WP2

Participants will mainly be recruited via 8–16 NHS participant identification centres (PICs) over 12 weeks. Clinicians and administrators at PICs will screen practice records to identify eligible young people who will be invited to express interest, and if selected, consented into the study. Convenience sampling techniques described for WP1 will also be used if needed, to achieve sample size.

Young people (n=30) will be ‘prescribed’ the web-app prototype then prompted to access it via one or two supported 1-to-2 sessions or group workshops, held online or in person, depending on preference. Participants will be invited to involve a supporter and provided with a link to the prototype for use between sessions. Pre- and post-measures will be collected via online survey platforms, trialling processes and measures to inform a future trial. A sub-sample of participants (n=up to 20) will complete think aloud interviews during sessions. Another sub-sample (n=14) will complete semi-structured interviews, to inform prototype development.

Healthcare professionals from the PICs, or recruited via broader study networks, will be invited to access the web-app via a supported session. They will provide qualitative data on their experiences signposting patients to the study and, if relevant, of engaging with the web-app via think aloud interviews and/or semi-structured interviews or focus groups.

### Outcomes measures and evaluation framework

Primary, secondary, qualitative and exploratory outcomes are outlined below. For a full matrix of data collection timepoints and outcome measures across work packages, see table 1.

**Table 1:** Data collection across work packages

Data type	Collection method	Measure	Approx. duration	Work package	Timepoint
Baseline and characterisation data					
Demographics	Online survey	Age, gender identity, ethnicity, education level, employment status. ADHD (self-reported diagnosis status, aged at diagnosis, treatment/support status). Single item literacy and digital literacy screeners^64^	5 min	1 and 2	Pre
ADHD symptom screener		Adult ADHD Self-Report Scale (ASRS v1.1) part A^65^	2 min	1 and 2	Pre
Primary outcome					
Acceptability	Online survey	Acceptability of Intervention Measure (AIM)^45^	2 min	1 and 2	Post
Secondary outcomes					
Knowledge, skills, confidence	Online survey	Patient Activation Measure (PAM)^46^	2 min	1 and 2	Post
User-experience		System usability scale^47^	2 min	1 and 2	Post
Qualitative evaluation and co-production					
User-experience	Teams/voice recorder	Think aloud interviews^66^ *Prompts during test (eg, first impressions, what thinking now, why choose?*) *Questions after test (eg, what you think, anything good/not good, what should be changed, use in NHS?*)	40 min	1 and 2	During
Young person perspectives		Semi-structured interview/focus group^59^ *Topics: experience of prototype engagement/research process. Reflections on the prototype, evaluation in NHS settings, tailoring to meet the needs of underserved groups, implementation*.	60 min	1 and 2	Post
Healthcare professional perspectives		Semi-structured interview/focus group *Topics: impressions of the prototype and experiences of ‘prescribing’ the prototype to patients. Barriers/facilitators to delivering a trial in NHS settings and sustainable implementation*	60 min	2	Post
Exploratory and implementation data					
Impairment	Online survey	Work and Social Adjustment Scale (WSAS)^50^	2 min	1 and 2	Pre post
Self-efficacy		General Self-Efficacy (GSE-6) scale^67^	2 min	1 and 2	Pre post
Self-compassion		Compassion Motivation and Action Scales: Self-Compassion (CMAS-self)^51^	5 min	2	Pre
Cost-effectiveness		Euroqol EQ-5D-5L^52^	4 min	2	Pre
User-experience	UX tool	Frequency and duration of app use	–	1 and 2	–
Feasibility of NHS implementation	Research records	Recruitment and retention rates	–	1 and 2	Post

ADHD, Attention deficit hyperactivity disorder; EQ-5D-5L, The EuroQol Organisation (EQ) 5 dimensions of health (5D), across 5 levels of severity (5L); UX, user experience.

#### Primary outcome

- Acceptability of the prototype: Measured post-intervention using the Acceptability of Intervention Measure^45^ (WP1 and 2). This brief psychometrically validated 4-item quantitative measure will provide data to indicate whether the digital tool is perceived as appropriate, appealing and relevant by the target population.

#### Secondary outcomes

- Patient activation: Measured post-intervention using the Patient Activation Measure^46^ (WP1 and 2). This brief, validated, quantitative measure is widely recognised within the UK healthcare ecosystem^8^ and directly evaluates the core behavioural mechanism of change of the intervention: increasing a young person’s underlying knowledge, skills and confidence in self-managing their long-term condition.
- Usability of prototype: Measured post-intervention using the System Usability Scale^47^ (WP1 and 2). This industry-standard reliable and sensitive 10-item scale of software usability can identify usability challenges and user satisfaction even within smaller sample sizes.^48 49^ Isolating technical software performance and user-interface challenges from the underlying clinical/educational impact of the intervention.

#### Qualitative and development outcomes

Young people’s interview topic guides will explore experiences of engaging with the prototype and the research, including feedback on the web-app, questionnaire burden and inclusion issues. Healthcare professional topic guides will explore their impressions of the prototype and reflections on ‘prescribing’ the prototype to patients. Also, barriers and facilitators to delivering a full trial within the NHS. Schedules and questionnaires will be finalised in consultation with stakeholders and the RAGs.

- Iterative prototype refinement (SmartADHD V.2): A co-developed web-app prototype and body of content, iteratively refined using data from think aloud and semi-structured interviews, paired with objective user experience analytics.
- Feasibility of NHS delivery: Evaluated qualitatively using interview and focus group data from young people and healthcare professionals (WP2) to map implementation barriers, implementation pathways and tailoring requirements for underserved groups.

#### Exploratory outcomes

- Feasibility of future effectiveness assessment: Evaluated via data completion rates and tracking changes across a range of pre/post exploratory measures. These include measures of functional impairment, via the *Work and Social Adjustment Scale*^50^ self-compassion tracking, via the Compassion Motivation and Action Scales^51^ and health economics parameters via the *Euroqol EQ-5D-5L.*^52^ Data compiled will inform power calculations and research design parameters for a future definitive trial (WP1 and 2).

### Data collection

This research will comply with the requirements of the UK General Data Protection Regulation (GDPR) and the Data Protection Act,^53^ with regard to the collection, storage, processing and disclosure of personal information and will uphold the Act’s core principles.

- Demographic and questionnaire data will be collected via certified, GDPR-compliant online survey tools (Qualtrics or Joint Information Systems Committee (JISC) Online Surveys).
- Think-aloud sessions and qualitative interviews will be recorded via secure, GDPR-compliant encrypted devices or secure software (eg, Microsoft Teams). A think-aloud schedule will guide sessions, drawing on templates from the Person-Based Approach^31^ and offering standard reflection prompts during the interaction and open questions afterwards.
- UX data (eg, session duration, page heatmaps, user interaction pathways) will be captured via data-secure UX tools integrated with Figma (eg, Maze or UXtweak).

### Analysis

Quantitative data will be managed and analysed using Stata SE17^54^ or Microsoft Excel.^55^ Descriptive statistics (frequencies, means, SD) will summarise baseline characteristics, as well as usability, acceptability and engagement metrics. In WP2, descriptive analyses will be extended to include recruitment and retention rates, data completeness patterns and self-compassion and cost-effectiveness metrics.^56^ To evaluate preliminary within-group changes and assess the practicalities of longitudinal data collection across the pre/post measures, paired-sample t-tests (or non-parametric equivalents) will be conducted.^42^ These will directly inform the power calculations and research design for a future definitive effectiveness-implementation trial.

Think Aloud data will be analysed following standard person-based-approach methods.^31^ Transcribed audio text will be sorted into a structured grid, organised by type (eg, content, layout, navigation) and separated into positive, neutral and negative feedback. Potential modifications to the prototype will be prioritised based on the MoSCoW (Must have, Should have, Could have, Won’t have) criteria^57^ and delivered in consultation with the research team and RAGs. The refinement process will be mapped and documented in a formal table of changes.

Qualitative data will be managed in NVivo^58^ and analysed using reflexive thematic analysis.^59 60^ A codebook approach will structure findings and provide insights suitable for applied policy research and NHS implementation.^59 61 62^ The process will be informed by acknowledgement of the researcher team’s active role in knowledge production and recognition of the interpretive nature of data coding.^59^ Generated themes will be checked by team members not directly involved in the analysis and members of the RAGs, to sense-check data interpretation.

Indicators of study burden (eg, measure completion times) and qualitative data on experiences of the research process will be synthesised to refine the subsequent trial blueprint. Final versions of the digital intervention will be locked in the conclusion of WP1 (C1 and P1) and WP2 (C2 and P2).

### Patient and public involvement

The research outlined in this protocol has evolved from engagement and co-production research undertaken in partnership with diverse stakeholders over the past 10 years. These include people with ADHD and their supporters, healthcare professionals working in primary and secondary care, commissioners and policy leads. For more information, please see the Science of ADHD and Neurodevelopment collaboration website, alongside publications arising from the Managing ADHD in Primary Care study.

PPIE forms a core part of delivery of this research and will be conducted in accordance with National Standards for Public Involvement,^63^ following principles of respect, support, transparency, fairness of opportunity and accountability. Engagement will be built in through the following structures:

- RAGs: meeting regularly to advise on research processes and planning, including:
  - Lived Experience RAG (LE-RAG): made up of 8–10 young people with ADHD and their supporters.
  - Healthcare Professional RAG (HP-RAG): made up of 8–10 health and social care professionals.
  - Digital Experts RAG (DE-RAG): made up of 2–8 digital experts from a range of sectors.
- Working groups (WGs): HP, LE and DE working groups will be convened, as needed, to provide input into prototype co-development. Working groups will provide flexible engagement opportunities, enhancing research inclusion for diverse young people with ADHD.

## Ethics and dissemination

###  Ethical approval and standards

This protocol has been approved by the Yorkshire and the Humber - Sheffield Research Ethics Committee (REC Reference: 26/YH/0101). The study will be carried out in accordance with the Declaration of Helsinki, the UK Policy Framework for Health and Social Care Research and the principles of Good Clinical Practice E6 (R3). All participants will be provided with a participant information sheet detailing the study’s purpose, risks and benefits prior to participation. Informed consent will be obtained digitally via an e-consent form before participants gain access to the study platform or complete any study procedures. Participants can withdraw from the study at any time without giving a reason and without affecting their standard care. The collaborative design will help to ensure that the research is conducted in a sensitive manner and results in outputs that are appropriately communicated and useful for this underserved population.

### Protocol amendments

Sponsor standard operating procedures are being followed for all amendments. It is anticipated that versions of study documents (eg, consent forms) may be updated following planned stakeholder engagement activities. Where documents are amended, Health Research Authority (HRA) guidance on amending an approval will be followed, and advice and approval of the study sponsor will be sought on whether changes qualify as non-substantial or substantial amendments. In all cases, HRA processes will be followed, with documents updated in Integrated Research Application System (IRAS) (using version control) and communicated to the REC.

### Dissemination and data sharing

Findings will be disseminated via research articles in peer-reviewed journals, conference presentations, public involvement events, patient groups and media releases. A summary of study findings will be shared with participants at the end of the study.
