# Supplementary material for: Co-development, initial evaluation and implementation of a digital health intervention (SmartADHD) for young people (aged 16–30) with attention deficit hyperactivity disorder, including optimisation for sustainable delivery via health and social care settings: protocol for a mixed-methods study
Source: BMJ Open. 2026 Oct 20;16(10):e127810. doi: 10.1136/bmjopen-2026-127810 (PMC13684235; doi:10.1136/bmjopen-2026-127810)
Supplement: Supplementary file 1 [file bmjopen-2026-127810.reviewer_comments.pdf]

## PEER REVIEW HISTORY

BMJ Open publishes all reviews undertaken for accepted manuscripts. Reviewers are asked to complete a checklist review form and are provided with free text boxes to elaborate on their assessment. These free text comments are reproduced below.

### ARTICLE DETAILS

**Title (Provisional)**

Co-development, initial evaluation, and implementation of a digital health intervention (SmartADHD) for young people (aged 16-30) with attention deficit hyperactivity disorder, including optimisation for sustainable delivery via health and social care settings: protocol for a mixed-methods study

**Authors**

Price, Anna; Bryant, Eleanor; Wenger, James; Groom, Madeleine J; Atherton, Helen; Newlove-Delgado, Tamsin

---

This article was not externally reviewed at BMJ Open. Protocols that have been independently assessed prior to submission to BMJ Open are usually fast-tracked to publication on the grounds that further substantial changes will not be possible. This independent assessment will usually be external, independent review for both a substantial grant award from a non-commercial or government funder and ethics approval.
